# The Anti-Inflammatory Properties of Terpenoids from *Cannabis*

**DOI:** 10.1089/can.2018.0014

**Published:** 2018-12-26

**Authors:** Ruth Gallily, Zhannah Yekhtin, Lumír Ondřej Hanuš

**Affiliations:** ^1^ The Lautenberg Center for General and Tumor Immunology, The Hadassah Medical School, The Hebrew University of Jerusalem, Jerusalem, Israel.; ^2^ Department of Medicinal and Natural Products, Institute for Drug Research, The Hadassah Medical School, The Hebrew University of Jerusalem, Jerusalem, Israel.

**Keywords:** cannabis, terpenoids, anti-inflammation, antinociceptive, CBD

## Abstract

**Introduction:** Cannabinoids are well known to have anti-inflammatory effects in mammalians; however, the Cannabis plant also contains other compounds such as terpenoids, whose biological effects have not yet been characterized. The aim of this study was to compare the anti-inflammatory properties of terpenoids with those of cannabidiol (CBD).

**Materials and Methods:** Essential oils prepared from three monoecious nonpsychoactive chemotypes of Cannabis were analyzed for their terpenoid content and subsequently studied pharmacologically for their anti-inflammatory properties *in vitro* and *in vivo*.

**Results:** *In vitro*, the three essential oils rich in terpenoids partly inhibited reactive oxygen intermediate and nitric oxide radical (NO^•^) production in RAW 264.7 stimulated macrophages. The three terpenoid-rich oils exerted moderate anti-inflammatory activities in an *in vivo* anti-inflammatory model without affecting tumor necrosis factor alpha (TNFα) serum levels.

**Conclusions:** The different Cannabis chemotypes showed distinct compositions of terpenoids. The terpenoid-rich essential oils exert anti-inflammatory and antinociceptive activities *in vitro* and *in vivo*, which vary according to their composition. Their effects seem to act independent of TNFα. None of the essential oils was as effective as purified CBD. In contrast to CBD that exerts prolonged immunosuppression and might be used in chronic inflammation, the terpenoids showed only a transient immunosuppression and might thus be used to relieve acute inflammation.

## Introduction

Human beings have used Cannabis or Cannabis products in various forms for thousands of years^1^ and references to therapeutic use of the plant are found in Hieratic script on papyri dated around 1700 BC.^2^ More recent reports have reviewed the history and characteristics of the materials^3^ and determined their clinical and biological properties.^4–7^ The Cannabis plant contains hundreds of different compounds apart from the major psychoactive compound Δ^9^-tetrahydrocannabinol (THC). Some of these are unique to the Cannabis plant,^8^ while others are shared with other members of the plant kingdom.

This century has seen a wealth of literature reports on the therapeutic potential of Cannabis and/or its constituents and a comprehensive review conducted by the Committee on the Health Effects of Marijuana: An Evidence Review and Research^9^ considered more than 10,700 relevant abstracts on this subject. They concluded that there was moderate to conclusive evidence for beneficial effects on chronic pain, and for a variety of other uses in different autoimmune and inflammatory diseases. While studies have focused on THC and the anti-inflammatory effects of the other major constituent, the nonpsychoactive cannabinoid cannabidiol (CBD),^10–14^ the effects of the aromatic terpene constituents have been largely neglected.^15^ Many of the terpenoids are of pharmacological values.^16^ About 200 terpenoids have been described in Cannabis and constitute the essential oil of the plant, being responsible for the characteristic odor of the Cannabis.^17^ The biochemical profiles of the terpenoids in a given plant are more closely associated to the genetics than the environment.^17,18^ Physiologically, they are responsible for protecting the plant from predators and attracting pollinating insects among other functions. Pharmacologically, they have been implicated in influencing the properties of the cannabinoids, possibly by a so-called entourage effect.^19^ Effects on anxiety have been noted as well as positive or negative influences on the antibacterial, anti-inflammatory, and sedative properties of Cannabis components.^19^ However, there is no consensus on the mechanism by which this is achieved and as to whether the terpenoids themselves possess pharmacologically significant properties.

We have previously demonstrated the ability of a triple assay, measuring swelling, pain, and tumor necrosis factor alpha (TNFα) serum titers, to measure the anti-inflammatory properties of CBD.^20,21^ In this study, we used a similar approach to investigate the antioxidant and anti-inflammatory properties of three different preparations of terpenoid-rich essential oils.

## Materials and Methods

### Essential oil samples

Samples rich in terpenoids were prepared from three monoecious nonpsychoactive chemotypes of hemp (legal in Europe). Tisza is a Hungarian variety, and Felina and Ferimon are chemotypes adapted to the climate in France.

All three chemotypes of Cannabis were harvested in August/September 2016 in the pre-Alpine region of Slovenia (Upper Savinja Valley), latitude NS 46°20′ 29.525 and longitude E 14°50′ 0.777. Samples of essential oil were prepared by steam distillation of female flowers (upper third of the plant).

### Terpenoid analysis

Samples (1 μL) of essential oil were analyzed by gas chromatography/mass spectrometry (GC/MS) in a Hewlett Packard G 1800B GCD system with an HP-5971 gas chromatograph, with an electron ionization detector. The software used was GCD Plus ChemStation and the column was an Rtx^®^ 5MS Low bleed GC/MS column (30 m×0.25 mm×0.25 μm film thickness). For analysis, the column was kept at 50°C for 4 min and then the temperature was programmed from 50°C to 280°C at 8°C/min; inlet 250°C; detector 280°C; splitless injection/purge time 1.0 min; initial temperature 100°C; and with initial time 4.0 min. The helium flow rate was 1 mL/min. Compound constituents were identified by comparison with standards and by the retention times, Kovats indices and by comparison with mass spectra from computerized libraries (HPCH2205, Wiley7N, and FENSC3).^22^ The terpenoids isolated by steam distillation as essential oil from each of the cannabis chemotypes gave the test samples T1 (Tisza chemotype), T2 (Felina chemotype), and T3 (Ferimon chemotype), whose analgesic and anti-inflammatory properties were characterized *in vitro* and *in vivo*.

### Cell culture

The murine monocyte/macrophage cell line RAW 264.7 (BALB/c) was obtained from the American Type Culture Collection (ATCC, Rockville, MD) and cultured in Dulbecco's modified Eagle's medium (DMEM) supplemented with 5% fetal calf serum (FCS), 1 mM sodium pyruvate and 100 μg/L streptomycin, and 100 IU/mL penicillin. The cell line is adherent and the cells were passaged by scraping from the culture dish.

### Reactive oxygen intermediate production

For reactive oxygen intermediate (ROI) assay, RAW 264.7 cells were removed from the culture dish by scraping, and were washed and resuspended at 10^6^ cells/mL in Hank's balanced salt solution without phenol red. Cells (5×10^5^) were added to a luminometer tube together with various concentrations of the essential oils (5, 10, 20, or 40 μg/mL). After 5 min, 10 μL luminol (Sigma) and 30 μL zymosan (Sigma) were added to each tube and the chemiluminescence was measured immediately in a luminometer (Biolumate LB 95; Berhold, Wilbad, Germany). A second set of samples was incubated for 24 h with the essential oils before adding luminol and zymosan. All experiments were done in duplicates.

### Nitric oxide (NO^•^) determination and MTT evaluation of viability

RAW 264.7 cells were seeded at a density of 1×10^5^ cells/well in 24-well plates and incubated overnight at 37°C and 5% CO_2_. On the following day, the medium was changed to fresh DMEM without FCS, containing various concentrations of the essential oils. The cells were then stimulated by the addition of lipopolysaccharide (LPS) to a concentration of 1 μg/mL. Cell supernatants (SNs) were harvested after 24 h for nitric oxide radical (NO^•^) assay by addition of 100 μL SN to an equal volume of Griess reagent (1% sulfanilamide, 0.1% naphthalene diamine, and 2% H_3_PO_4_). After 10 min of incubation, the resultant color was measured at 550 nm. The amount of NO^•^ produced, and any inhibition by the test materials, was calculated from a standard curve prepared with NaNO_2_.

The viability of the cells after incubation with the test materials was determined by MTT viability staining. The absorbance was measured at 550 nm on a microplate reader.

### Animals

Female Sabra mice (Israel), 7–8 weeks old, were maintained in the specific-pathogen-free unit of the Hadassah Medical School, Hebrew University, Jerusalem, Israel. The experimental protocols were approved by the Institutional Animal Care Ethics Committee. The animals were maintained at a constant temperature (20–21°C) and a 12-h light/12-h dark cycle, and were provided a standard pellet diet with water *ad libitum*.

### Induction and treatment of paw inflammation

Inflammation was induced by injection of 40 μL of a suspension of 1.5% w/v zymosan A (Sigma) in saline into the subplanter surface of the right hind paw of the mice. This was followed immediately by an injection of the sample intraperitoneally (10, 25, or 50 mg/kg). For injection, the terpenoids were dissolved in vehicle containing ethanol:Cremophore:saline at a ratio of 1:1:18. CBD was used as a positive control. Paw swelling and pain perception were assessed after 2, 6, and 24 h. Blood was collected after 24 h for analysis of TNFα serum levels.

### Evaluation of edema

Calibrated calipers were used to measure paw swelling (thickness) 2, 6, and 24 h after injection of zymosan.^20^

### Pain assay

Pain at 2, 6, and 24 h after zymosan injection was assessed by the von Frey nociceptive filament assay,^23^ where 1.4–60 g filaments, corresponding to 4.17–5.88 log of force, was used to test the sensitivity of the swollen paw. The untreated hind paw served as a control. The measurements were performed in a quiet room and the animals were handled for 10 s before the test. A trained investigator then applied the filament, poking the middle of the hind paw to provoke a flexion reflex, followed by a clear finch response after paw withdrawal. Filaments of increasing size were each applied for about 3–4 s. The mechanical threshold force in grams was defined as the lowest force required to obtain a paw retraction response.

### Measurement of TNFα

Blood was collected 24 h after zymosan injection, and the sera were assayed for TNFα using a mouse TNFα ELISA kit (R&D System), according to the manufacturer's instructions.

### Statistical analysis

Statistical calculations used the nonparametric Mann-Whitney *U* test and Wilcoxon signed-rank test. The results are presented as average±standard error.

## Results

### The terpenoid content of essential oils from three different Cannabis chemotypes

**FIG. 1.**
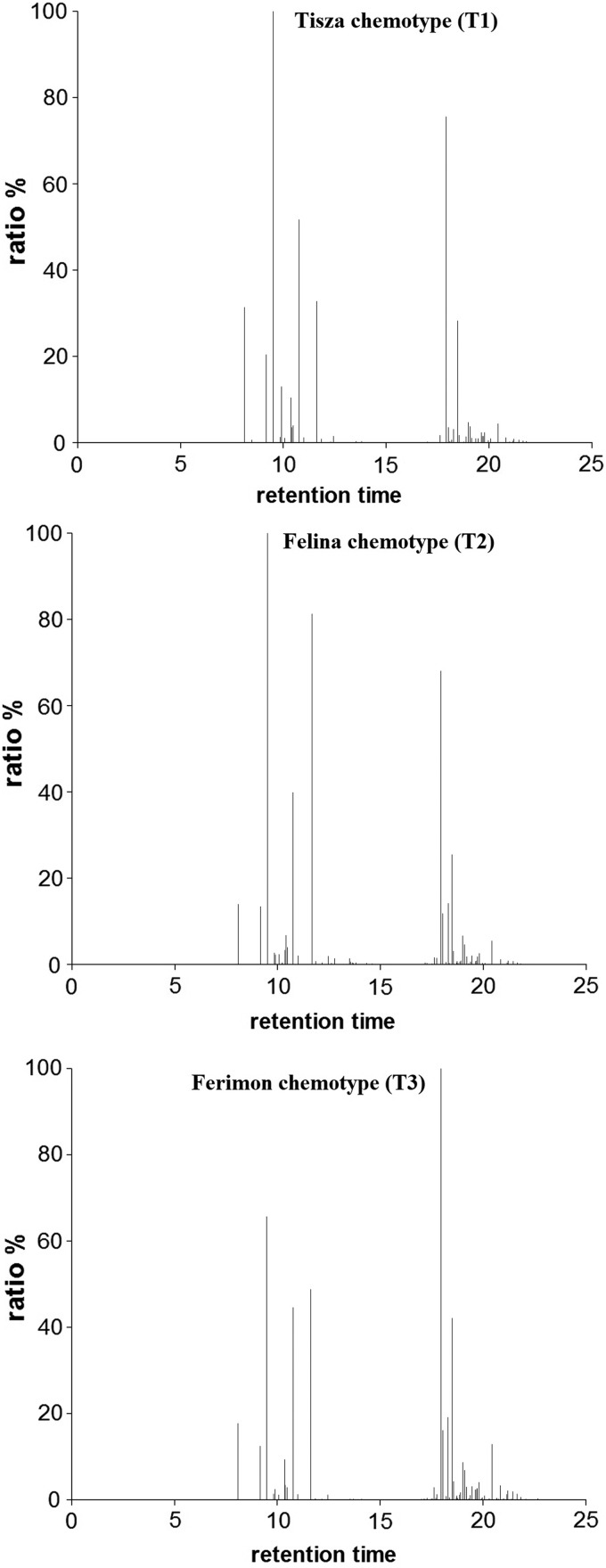
GC/MS spectra of essential oils from three different chemotypes of Cannabis—Tisza (T1), Felina (T2), and Ferimon (T3). Each chemotype displays an individual, characteristic profile. The identity of each peak is summarized in Table 1. GC/MS, gas chromatography/mass spectrometry.

The essential oils from each of the three different chemotypes of Cannabis—Tisza (T1), Felina (T2), and Ferimon (T3)—were analyzed by GC/MS analyses, and up to 50 different compounds were identified (Fig. 1 and Table 1). The spectra show similarities and differences between the different Cannabis chemotypes. As the amounts of the identified terpenoids were not quantified, the results in Table 1 are presented as the relative ratio to the main terpene in the sample, which was set to 100.00%.

**Table 1.** Terpenoid Content in the Three Chemotypes of Cannabis: The Results Are Presented as the Relative Ratio to the Main Terpene in the Sample, Which Was Set to 100.00%

Terpene	Kovats Index	Tisza chemotype (T1) %	Felina chemotype (T2) %	Ferimon chemotype (T3) %
α-Pinene	5.85	31.420	14.007	19.413
Camphene	6.26	0.640	—	0.108
β-Pinene	7.04	20.461	13.434	10.153
Myrcene	7.43	100.000	100.000	52.755
α-Phellandrene	7.85	1.316	2.693	1.163
Δ^3^-Carene	8.10	13.020	2.240	2.103
α-Terpinene	8.30	1.094	2.376	1.018
*o*-Cymene	8.59	—	0.221	0.564
*p*-Cymene	8.53	0.105	0.396	—
Limonene	8.69	10.482	3.379	7.142
β-Phellandrene	8.70	3.537	6.794	2.668
*cis*-β-Ocimene	8.96	4.043	4.005	2.520
*trans*-β-Ocimene	9.42	51.700	39.864	32.634
γ-Terpinene	9.78	1.151	2.041	0.880
Terpinolene	10.98	32.842	81.256	38.728
Linalool	11.32	0.905	—	—
1,3,8-*para*-Menthatriene	11.86	—	0.449	0.377
*endo*-Fenchol	12.10	0.263	—	—
*allo*-Ocimene	12.70	1.517	1.918	1.179
Terpinen-4-ol	14.66	0.294	0.665	
*p*-Cymen-8-ol	14.91	—	0.421	
Hexyl butanoate	15.40	—	0.362	
α-Terpineol	15.21	0.303	0.466	
Eugenol	22.70	—	—	1.199
α-Ylangene	23.43	0.191	0.148	0.205
α-Copaene	23.49	—	0.144	0.204
Hexyl hexanoate	23.83	—	0.411	—
7-*epi*-Sesquithujene	24.19	—	0.288	0.222
Sesquithujene	24.84	—	0.231	—
*cis*-Caryophyllene	24.95	1.778	1.669	2.072
*cis*-α-Bergamotene	25.10	0.148	1.517	0.846
*trans*-Caryophyllene	25.36	75.569	68.099	100.000
*trans*-α-Bergamotene	25.99	3.578	11.867	11.100
α-Guaiene	26.20	0.387	—	—
*trans*-β-Farnesene	26.92	3.157	14.176	11.811
α-Humulene	26.82	28.338	25.453	33.158
*allo*-Aromadendrene	27.07	1.744	3.130	—
*ar*-Curcumene	27.96	—	0.310	0.323
β-Selinene	28.37	4.745	6.653	6.007
α-Selinene	28.74	3.785	4.654	4.747
*cis*-α-Bisabolene	29.09	—	—	1.719
*trans*-α-Bisabolene		1.524	—	—
δ-Cadinene	29.72	—	0.621	—
β-Sesquiphellandrene	29.70	—	2.011	—
Selina-3,7(11)-diene	30.66	2.343	2.577	2.813
Caryophyllene oxide	32.16	4.437	5.523	—
Humulene epoxide II	33.20	1.151	1.176	—
*allo*-Aromadendrene epoxide	34.42		0.426	—
α-Bisabolol	36.17		0.226	—

### *In vitro* studies

#### Suppression of ROI and NO^•^ production by RAW macrophages incubated with the terpenoid-rich essential oils

**FIG. 2.**
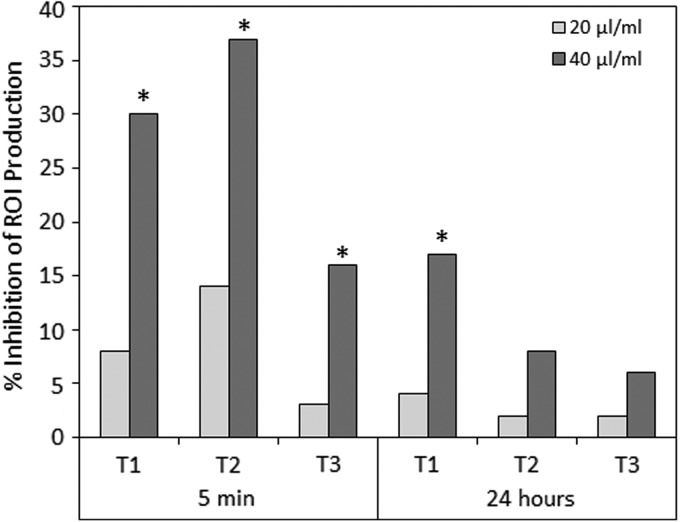
Zymosan-induced generation of ROIs by RAW 264.7 macrophages was inhibited by essential Cannabis oils from each of the three chemotypes Tisza (T1), Felina (T2), and Ferimon (T3). RAW 264.7 macrophages (5×10^5^/500 μL HBSS) were either untreated (Control) or incubated with 20 or 40 μL essential oils for 5 min or 24 h before ROI induction by zymosan. The ROI was measured by luminol chemiluminescence. The percentage inhibition of ROI production is presented. *p<0.05. ROI, reactive oxygen intermediate; HBSS, Hank's Balanced Salt Solution.

To study the effects of terpenoids on essential macrophage functions, the RAW 264.7 macrophage cell line was either untreated or incubated with the essential oils at indicated concentrations, before stimulation with zymosan to induce ROIs or LPS to induce NO^•^ production. The ROI production was measured by luminol chemiluminescence, while NO^•^ production was measured by resulting nitrite concentration in the supernatant. The generation of ROI by RAW 264.7 macrophages was significantly suppressed following a short 5 min-incubation with 40 μg/mL terpenoids from chemotypes T1 and T2 (Fig. 2), while lower concentrations had barely any effect. T3 terpenoids, however, showed only a moderate inhibition at 40 μg/mL (Fig. 2). When the macrophages were incubated with terpenoids for 24 h before zymosan induction of ROI, the terpenoids had barely any inhibitory effect (Fig. 2). This observation suggests for a transient inhibitory effect of terpenoids.

**FIG. 3.**
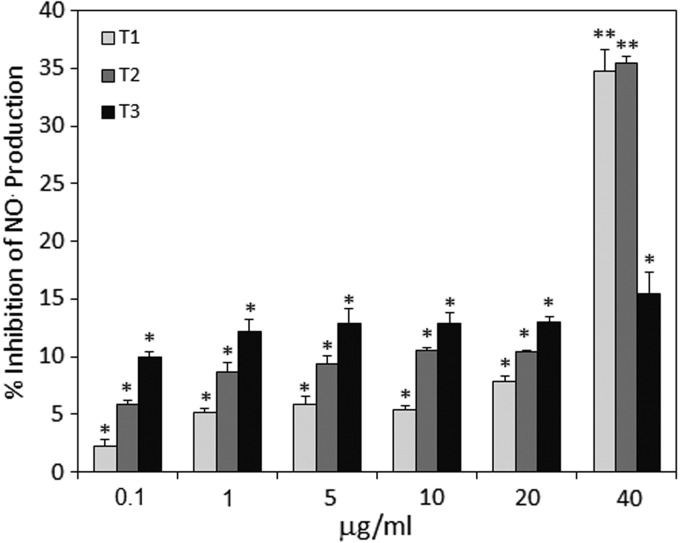
LPS-induced generation of NO^•^ by RAW 264.7 macrophages was inhibited by essential Cannabis oils from each of the three chemotypes Tisza (T1), Felina (T2), and Ferimon (T3). RAW 264.7 macrophages were incubated in serum-free medium alone or in the presence of various amounts of the essential oils, as indicated in the figure. After 5 min, the macrophages were exposed to LPS (1 μg/mL) for 24 h and the nitrite concentration in the supernatant reflecting NO^•^ production was measured using the Griess reagent. *p<0.05, **p<0.01. LPS, lipopolysaccharide.

Similar to ROI inhibition, the T1 and T2 essential oils significantly suppressed LPS-induced NO^•^ production by RAW macrophages when applied at a concentration of 40 μg/mL (Fig. 3). Lower concentrations of T1 and T2 had almost no effect. The T3 essential oil had barely any effect at the concentrations used.

The MTT assay showed that the inhibition of NO^•^ and ROI by terpenoids was not due to cytotoxicity, since all the cells remained over 80% viable with all concentrations tested (data not shown).

### *In vivo* studies

#### Anti-inflammatory and antinociceptive effects of terpenoid-rich essential oils

**FIG. 4.**
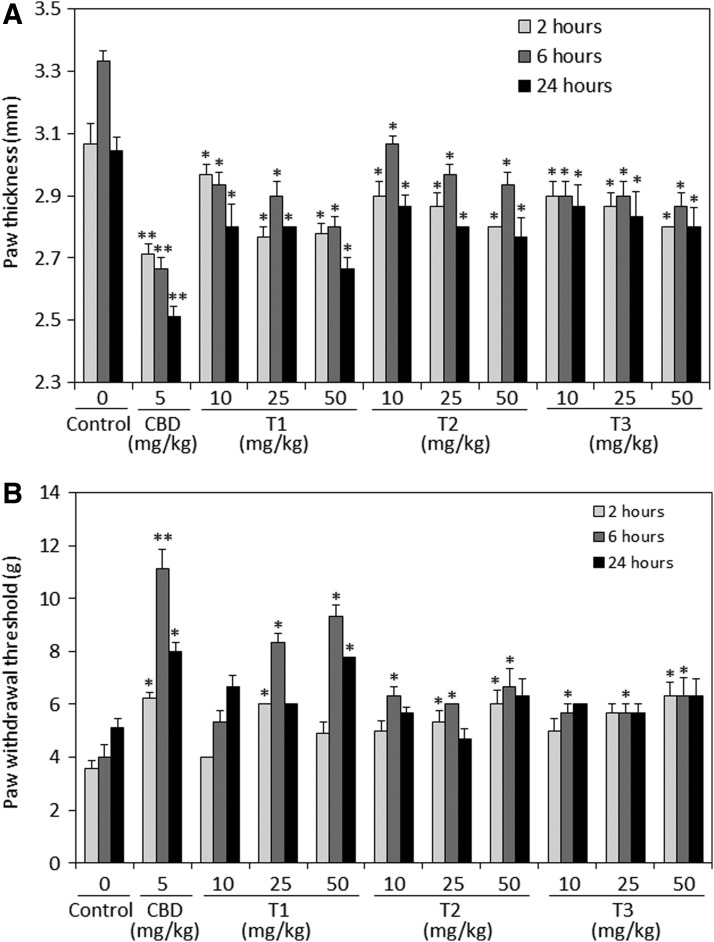
Anti-inflammatory **(A)** and antinociceptive **(B)** effects of intraperitoneally injected CBD or essential oils from each of the three Cannabis chemotypes Tisza (T1), Felina (T2), and Ferimon (T3). **(A)** Prevention of zymosan-induced swelling of hind paw; 1.5% zymosan in 40 μL was injected into the subplanter surface of the right hind paw. Immediately thereafter, CBD (5 mg/kg) or essential oils (10, 25, or 50 mg/kg) dissolved in vehicle containing ethanol:Cremophore:saline at a ratio of 1:1:18 was injected intraperitoneally. The paw thickness indicative for paw swelling was measured 2, 6, and 24 h thereafter. The paw thickness of untreated mice was 2.3 mm, which made the baseline of the graph. *N*=9 in each treatment group. *p<0.05, **p<0.01. **(B)** The hyperalgesia occurring after zymosan injection in control and treated mice as described in **(A)** was measured by using the von Frey nociceptive filament assay. The higher the paw withdrawal threshold, the higher is the antinociceptive effect of the drug. *N*=9 in each treatment group. *p<0.05, **p<0.01. CBD, cannabidiol.

In this study, we used the well-accepted mouse model of zymosan-induced inflammation to investigate the anti-inflammatory and antinociceptive activities of the three terpenoid preparations. The extent of hind paw swelling was determined 2, 6, and 24 h following paw injection of 60 μg zymosan alone (control) or together with intraperitoneal injection of various concentrations of essential oils from each of the three chemotypes Tisza (T1), Felina (T2), and Ferimon (T3). For comparison, a mouse group was treated with CBD (5 mg/kg), which is well known to exert anti-inflammatory and antinociceptive effects.^20,21^ Intraperitoneal injection of each of the three terpenoid preparations significantly reduced zymosan-induced paw swelling at all three concentrations tested (10, 25, and 50 mg/kg) (Fig. 4A). There were no significant differences between the three concentrations, meaning that a plateau effect has been reached and no further inhibition can be achieved by increasing the dose. Also, it should be noted that the three terpenoids were less potent than CBD.

Next, we studied the antinociceptive effects of the three terpenoid preparations in comparison to CBD. To this end, the same mice described above for zymosan-induced paw swelling were used to determine the paw withdrawal threshold by applying von Frey filaments on the paws. Higher paw withdrawal threshold is indicative for better pain-relieving effects. As expected, CBD significantly increased the paw withdrawal threshold (Fig. 4B). Also, the T1 terpenoids at 25 and 50 mg/kg could significantly increase the pain threshold (Fig. 4B), although less potent than CBD. T1 showed a correlative dose–response at 6 h, while no significant difference between the three dose groups could be observed at 2 and 24 h (Fig. 4B).

In contrast, only a moderate pain inhibition could be achieved with the T2 and T3 terpenoid preparations (Fig. 4B). No correlative dose–response could be seen for T2 and T3, suggesting for having reached a maximum effect. The more potent pain-relieving effects of T1 in comparison to T2 and T3 is correlative to the better prevention of paw swelling by T1 (compare Fig. 4B with 4A). Of note, the antinociceptive effects of all compounds, including CBD, were most prominent at 6 h.

#### TNFα serum titer

**FIG. 5.**
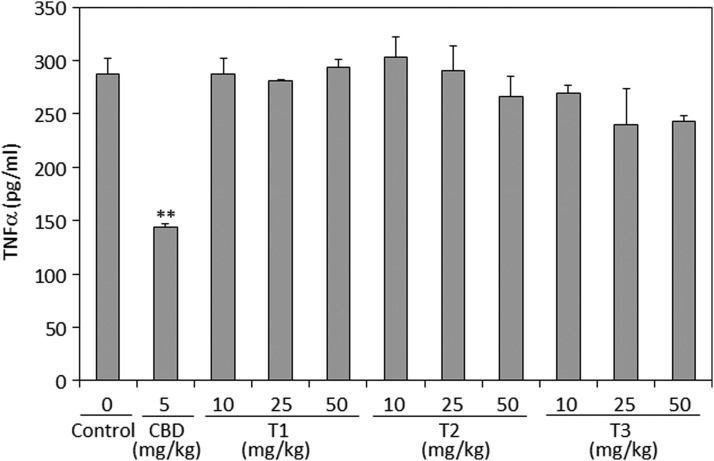
TNFα in the sera of mice treated with zymosan and essential oils. Twenty-four hours after injecting zymosan and/or an intraperitoneal dose of CBD (5 mg/kg) or essential oils (10, 25, or 50 mg/kg) dissolved in vehicle containing ethanol:Cremophore:saline at a ratio of 1:1:18, the TNFα concentration in the serum was determined by ELISA. *N*=3 for each treatment group. **p<0.01. TNFα, tumor necrosis factor alpha.

TNFα is one of the proinflammatory cytokines that is produced during inflammation and activates the nociceptive terminals that innervate the inflamed tissue.^24^ It was therefore important to study the effect of terpenoids on TNFα serum levels. However, none of the terpenoid preparations had any significant effect on the TNFα serum level 24 h after zymosan injection (Fig. 5). Under the same conditions, CBD reduced the level of TNFα significantly by about 48% (Fig. 5).

## Discussion

The terpenoids provide the cannabis plant with its characteristic fragrance and it is generally accepted that they provide protection from marauding insects. Although more than 230 different named terpenoids have been identified, in Cannabis, only about 50 known terpenoids have been identified in a single plant sample, and the profile may be characteristic of a given chemotype (Hanuš LO, unpublished data). This variety is reflected in the differences noted between the three cannabis chemotypes used in this study. Despite suggestions that differences in the pharmaceutical properties of different chemotypes may be a consequence of the variety of terpenoids present, there is almost no information about the biological and medical properties of cannabis-derived terpenoids.

We have previously developed a triple assay to demonstrate the anti-inflammatory and antinociceptive properties of CBD.^20,21^ This assay measures the ability of any compound to inhibit zymosan-induced paw swelling and to relieve zymosan-induced pain. In addition, by collecting blood 24 h after zymosan injection, the assay enables us to determine the effects of the compounds on zymosan-induced TNFα production. We adapted this method to study the anti-inflammatory properties of terpenoid-rich essential oils from three different chemotypes of Cannabis.

Our data show that the three essential oils, which contain various ratios of 48 identified terpenoids, show moderate anti-inflammatory properties in an induced paw swelling model in mice. All three preparations were much less potent than CBD. Also, no correlative dose–response was observed, suggesting that a maximum effect was observed already with the lower dose. T2 was somewhat less potent than T1 and T3 with regard to their paw swelling inhibitory effects. T1, but not T2 and T3, exhibited moderate antipain effects, but still, T1 was less potent than CBD. The differences between terpenoids and CBD might be explained by their different effect on TNFα production. While CBD strongly reduces TNFα production *in vivo*, the terpenoids barely had any effect. *In vitro*, the terpenoids only affected macrophage functions such as ROI and NO^•^ production at high concentration (40 μg/mL), which is in contrast to 6 μg/mL CBD required to inhibit 90% of granulocyte-induced ROI production^25^ and 8 μg/mL CBD to inhibit 50% of zymosan-induced ROI in RAW 264.7 macrophages.^21^

The effects were chemotype specific to a certain extent, which is in agreement with the individuality of the essential oils with terpenoids. Interestingly, in contrast to CBD, none of the chemotype essential oil had any effect on the levels of zymosan-induced TNFα. This might suggest the terpenoids exert their anti-inflammatory effects through a mechanism other than that employed by the cannabinoids.

## Conclusions

Different chemotypes of cannabis have a distinctive composition of terpenoids. These essential oils do have anti-inflammatory and antinociceptive activities that vary according to their composition, but they had no effect on TNFα titers. None of the essential oils was as effective as CBD. We suggest that terpenoids may be used to diminute acute inflammation effect, whereas the cannabinoids to inhibit chronic inflammation symptoms.

## Abbreviations Used

CBD: cannabidiol
DMEM: Dulbecco's modified Eagle's medium
FCS: fetal calf serum
GC/MS: gas chromatography/mass spectrometry
KI: Kovats Index
LPS: lipopolysaccharide
NO: nitric oxide
ROI: reactive oxygen intermediate
SNs: supernatants
THC: tetrahydrocannabinol
TNFα: tumor necrosis factor alpha
